# Supplementary material for: The influence of cortical activity on perception depends on behavioral state and sensory context
Source: Nat Commun. 2024 Mar 19;15:2456. doi: 10.1038/s41467-024-46484-5 (PMC10951313; doi:10.1038/s41467-024-46484-5)
Supplement: Supplementary file 1 — Supplementary Information [file 41467_2024_46484_MOESM1_ESM.pdf]

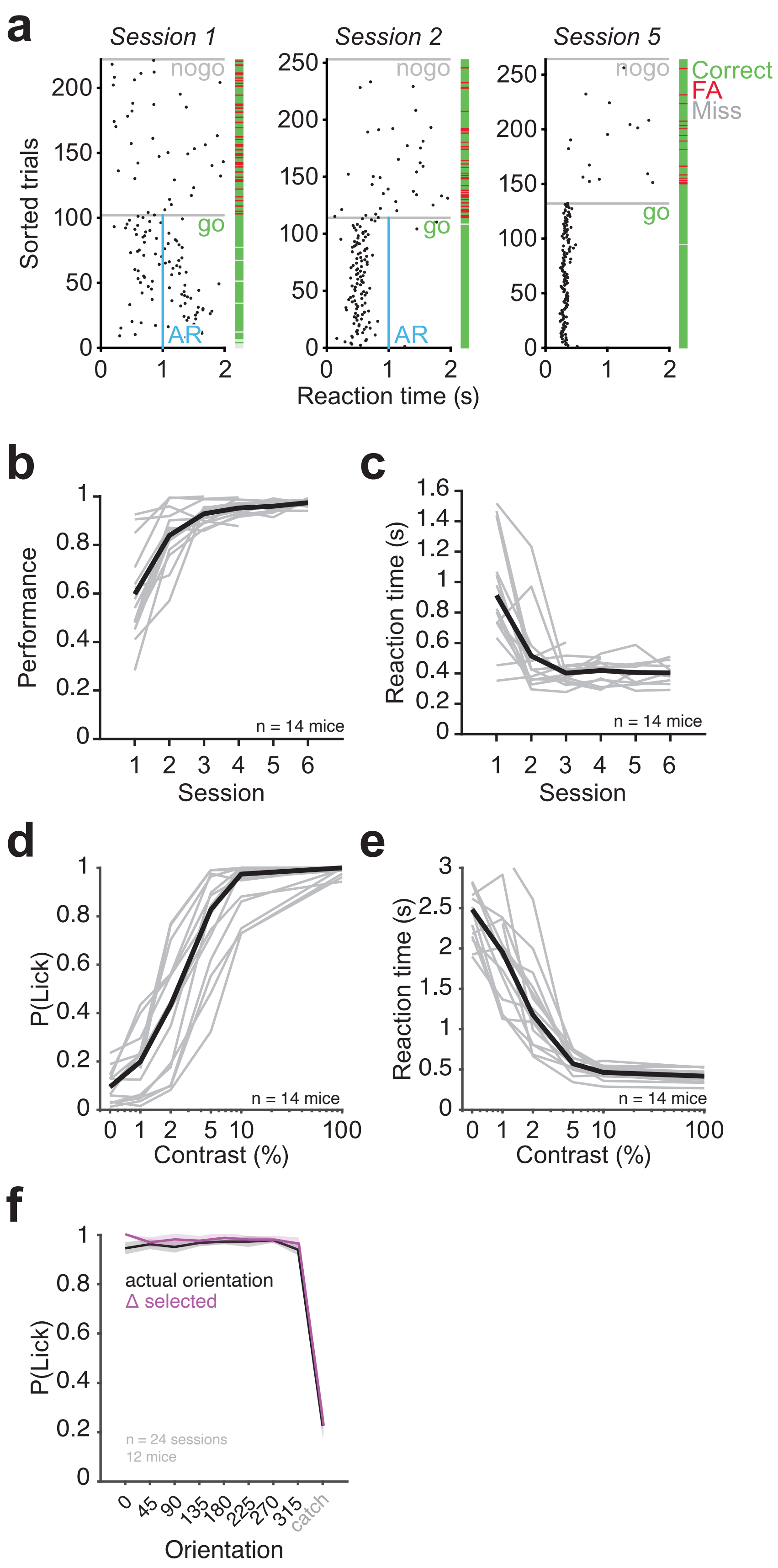
a
Session 1
Session 2
Session 5
nogo
go
AR
Correct
FA
Miss
Sorted trials
Reaction time (s)
b
Performance
Session
n = 14 mice
c
Reaction time (s)
Session
n = 14 mice
d
P(Lick)
Contrast (%)
n = 14 mice
e
Reaction time (s)
Contrast (%)
n = 14 mice
f
P(Lick)
actual orientation
Δ selected
n = 24 sessions
12 mice
Orientation
catch

Supplementary Figure 1. Task acquisition

a) Example lick raster plots, sorted by trial type (go and catch) for the first, second and fifth day of behavioral training for one example animal. Black dots indicate the first lick (reaction time) after presentation of the visual stimulus. Blue line indicates the timing of the automatically delivered water reward (AR) in the early training sessions.
b) Performance improves across training sessions. Performance is defined as the probability of the first lick occurring before the automatic reward delivery.
c) Reaction times decrease as the mice learn the task.
d) In trained animals the stimulus detection probability decreases as the contrast decreases.
e) In trained animals the reaction times increase as the contrast decreases.
f) In trained animals during the final experiment there is no behavioral bias towards the stimulus orientation chosen (to match the preference of the cotuned stimulation ensemble).

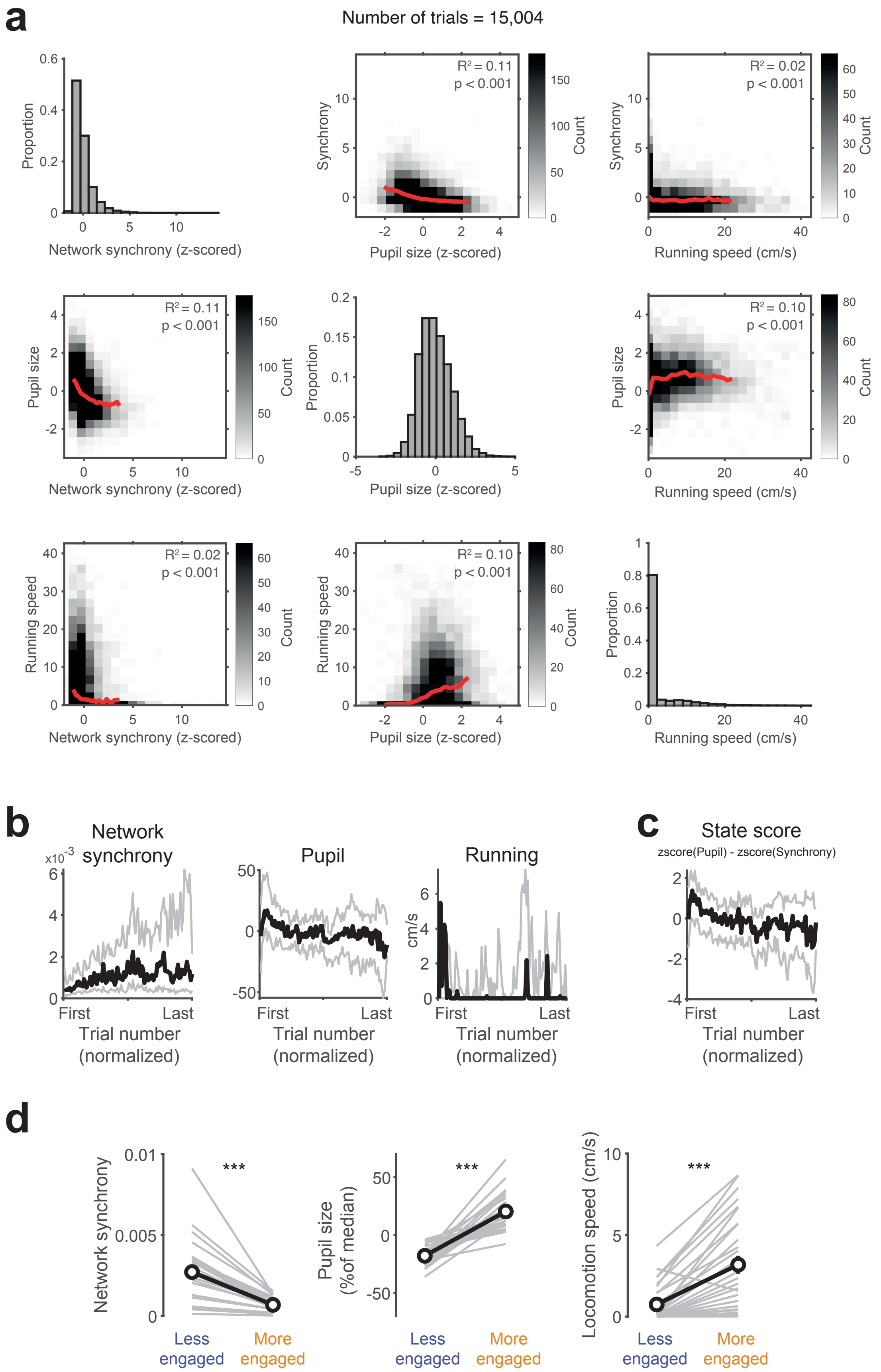

Supplementary Figure 2

Supplementary Figure 2. Relationship between state variables and evolution during the behavioral session

a) Plot matrix where the diagonal describes the distribution of 3 key behavioral state measures, all computed in the same pre-trial window: neuronal synchrony, pupil size and locomotion. All trials from all sessions pooled. The off-diagonals describe the pairwise relationships between all sets of two of the three measures. Greyscale shading represents number of trials in that bin. Red line indicates the binned average. Statistics indicate Pearson correlation. Note the relationship between pupil size and neuronal synchrony. Note the majority of trials are stationary not during locomotion. n = 14 mice, 28 sessions.
b) The change in behavioral state measure across trials, with normalized session length to facilitate averaging across sessions. Black line represents the median, and Grey lines indicate 25th and 75th percentiles across sessions. Note the low value of running speed – most trials are not associated with locomotion.
c) Same as b but the z-scored combination of neuronal synchrony and pupil size (used to classify trials into the more or less engaged states) across trials.
d) The average neural synchrony, pupil size and locomotion speed in the more/less engaged states used throughout the study. Note the low running speeds – most trials are associated with no locomotion.

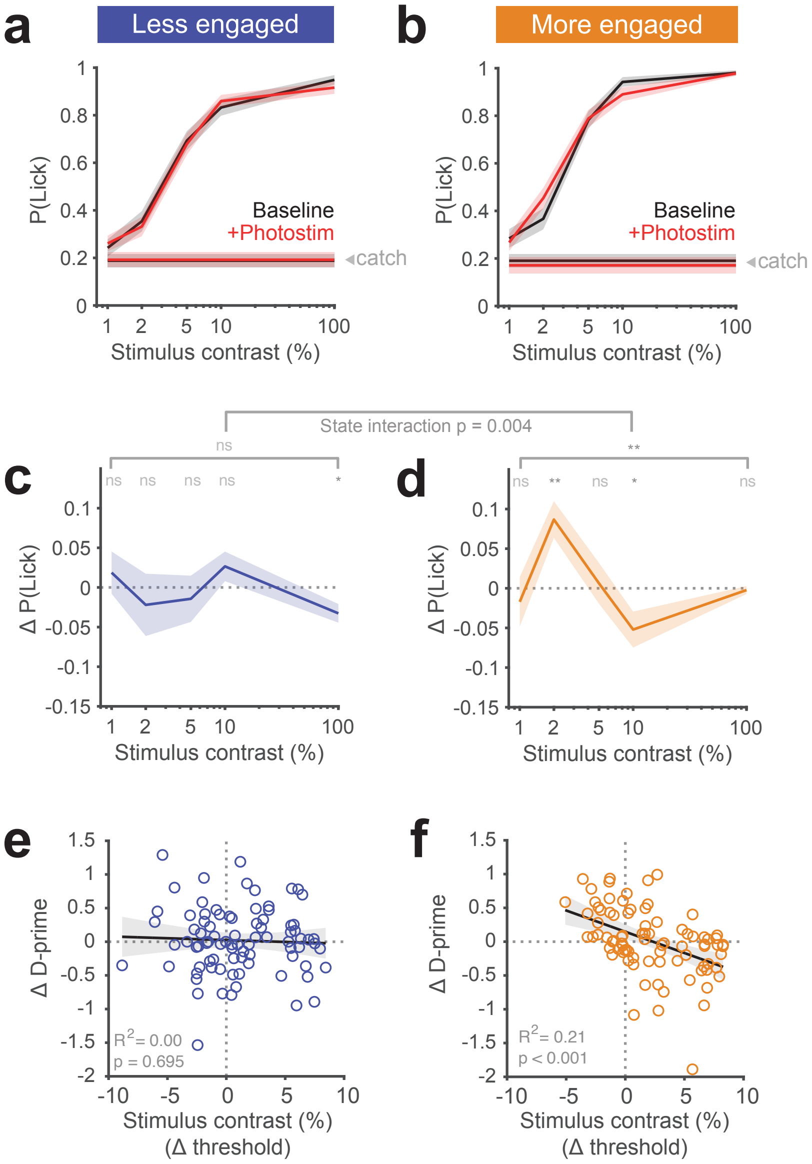
a
Less engaged
b
More engaged
P(Lick)
Baseline
+Photostim
catch
Stimulus contrast (%)
State interaction p = 0.004
ns
**
c
d
ns ns ns ns *
ns ** ns * ns
Δ P(Lick)
Stimulus contrast (%)
e
f
Δ D-prime
R2= 0.00
p = 0.695
R2= 0.21
p < 0.001
Stimulus contrast (%)
(Δ threshold)

Supplementary Figure 3. Photostimulation during the engaged state increases licking to lower contrast stimuli and decreases licking to higher contrast stimuli

a) Probability of licking to visual stimulus without (black) or with (red) photostimulation during the less engaged behavioral state. Horizontal line represents the 0% visual stimulus. n = 14 mice, 28 sessions.
b) Same as a) but for the more engaged behavioral state.
c) The photostimulation-mediated change in licking during the less engaged state. P-value above the plot indicates the ANOVA result for a difference amongst the contrasts. P-value above each contrast indicates a significant change on that contrast.
d) Same as c) but for the more engaged behavioral state.
e) Relationship between the photostimulation-mediated change in behavior and the stimulus contrast aligned to that session's perceptual threshold. Only threshold adjacent trials (1% and 100% excluded) are shown. Each point indicates the average change in behavior at one contrast in one session.
f) Same as for e) but for the more engaged behavioral state. Note the relationship between stimulus contrast (aligned to threshold stimulus) and the photostimulation-mediated change in behavioral report. In a combined regression model (including the behavioral changes during both less-engaged and more-engaged states) model there is a significant interaction of the engagement state term ('less or 'more) with the threshold-aligned stimulus contrast.

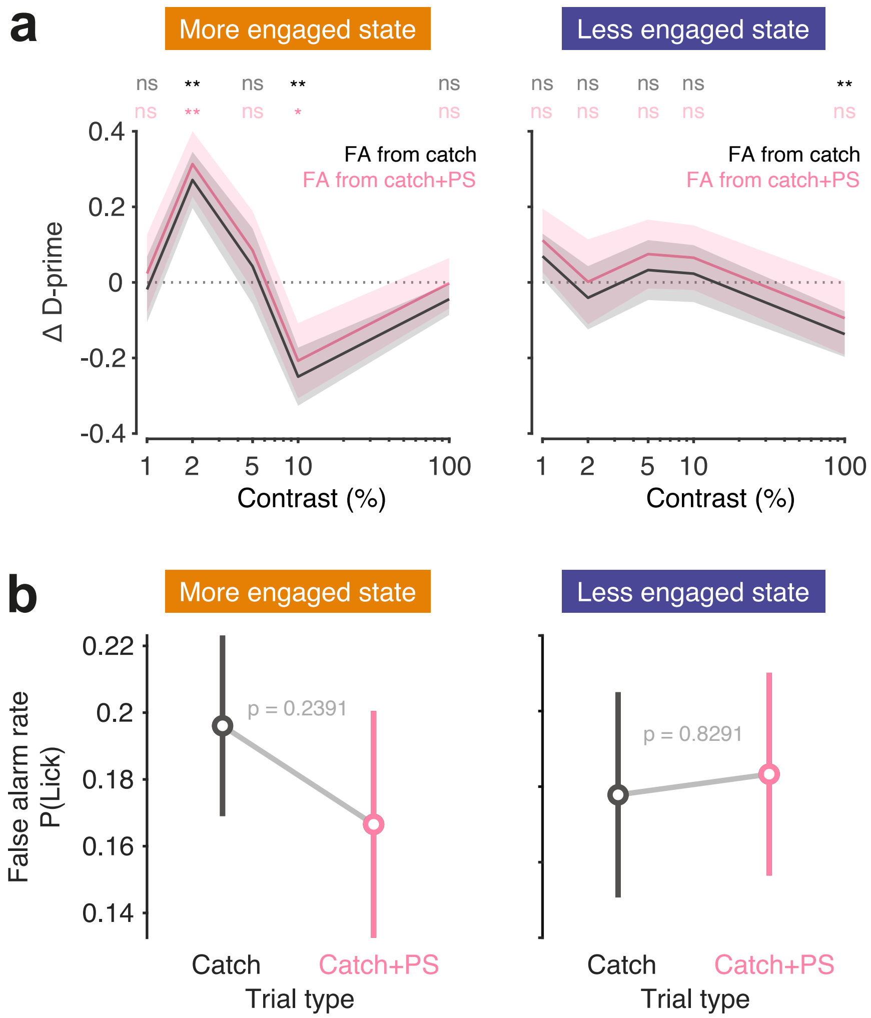
a
More engaged state
Less engaged state
ns ** ns ** ns
ns ** ns * ns
ns ns ns ns **
ns ns ns ns ns
FA from catch
FA from catch+PS
Δ D-prime
0.4
0.2
0
-0.2
-0.4
1 2 5 10 100
Contrast (%)
b
False alarm rate P(Lick)
0.22
0.2
0.18
0.16
0.14
p = 0.2391
p = 0.8291
Catch
Catch+PS
Trial type

Supplementary Figure 4. D-prime calculation using hit and false alarm rates from photostimulation trials

a) Left, change in behavior induced by photostimulation as a function of contrast for the more engaged state. Right, same for the less engaged state. Black line, d-prime calculation using false alarm rate from catch trials. Pink line, using false alarm rate from catch+photostimulation trials. n = 14 mice, 28 sessions. Significance stars indicate results of point-wise one-sample Wilcoxon rank sum test with Bonferroni correction.
b) Left, false alarm rates used in the two alternative methods of d-prime calculation for the engaged state. Right, same for the less engaged state. Paired Wilcoxon signed-rank test.

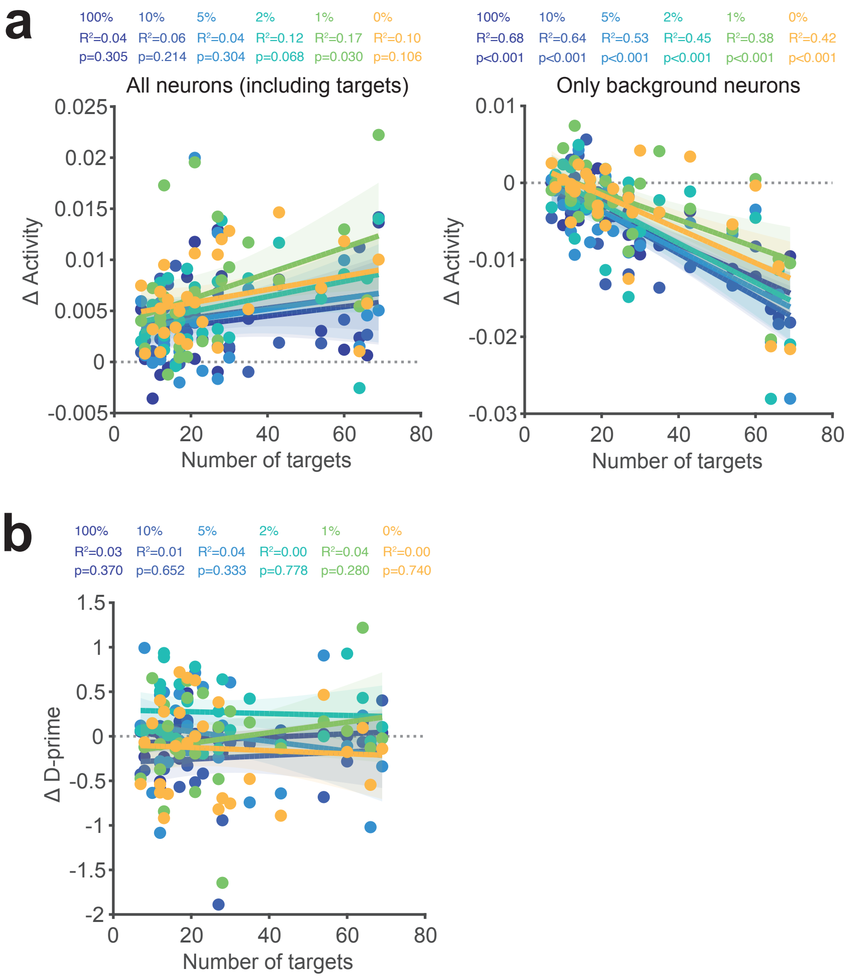

Supplementary Figure 5

Supplementary Figure 5. Increasing the number of stimulated cells progressively suppresses more of the background population, but has no effect on behavior

a) The average photostimulation-mediated change in activity in the target cells (left) and the background cells (right) as a function of number of photostimulated cells. Each datapoint represents the average activity change during all trials of one contrast in one session (no difference between states observed, states are pooled for simplicity). There is a strong increase in the level of background suppression as the number of stimulated cells increases.

b) There is no relationship between number of stimulated cells and the magnitude of the behavioral change. We suggest this is because of the balance between target activation and background suppression, acting to normalize activity levels.

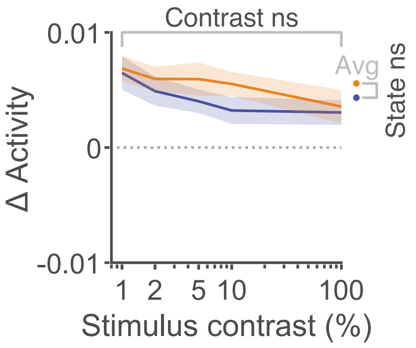
Contrast ns
0.01
0
-0.01
Δ Activity
Avg
State ns
1
2
5
10
100
Stimulus contrast (%)

Supplementary Figure 6. No effect of contrast on the average total change in activity following photostimulation

There is no difference in change of activity across all cells (targets and background) following photostimulation during any of the visual stimulus contrasts (2-way ANOVA main effect of contrast $F = 2.1$, $p = 0.082$, main effect of state $F = 0.2$, $p = 0.913$, $n = 28$ session (14 mice)).

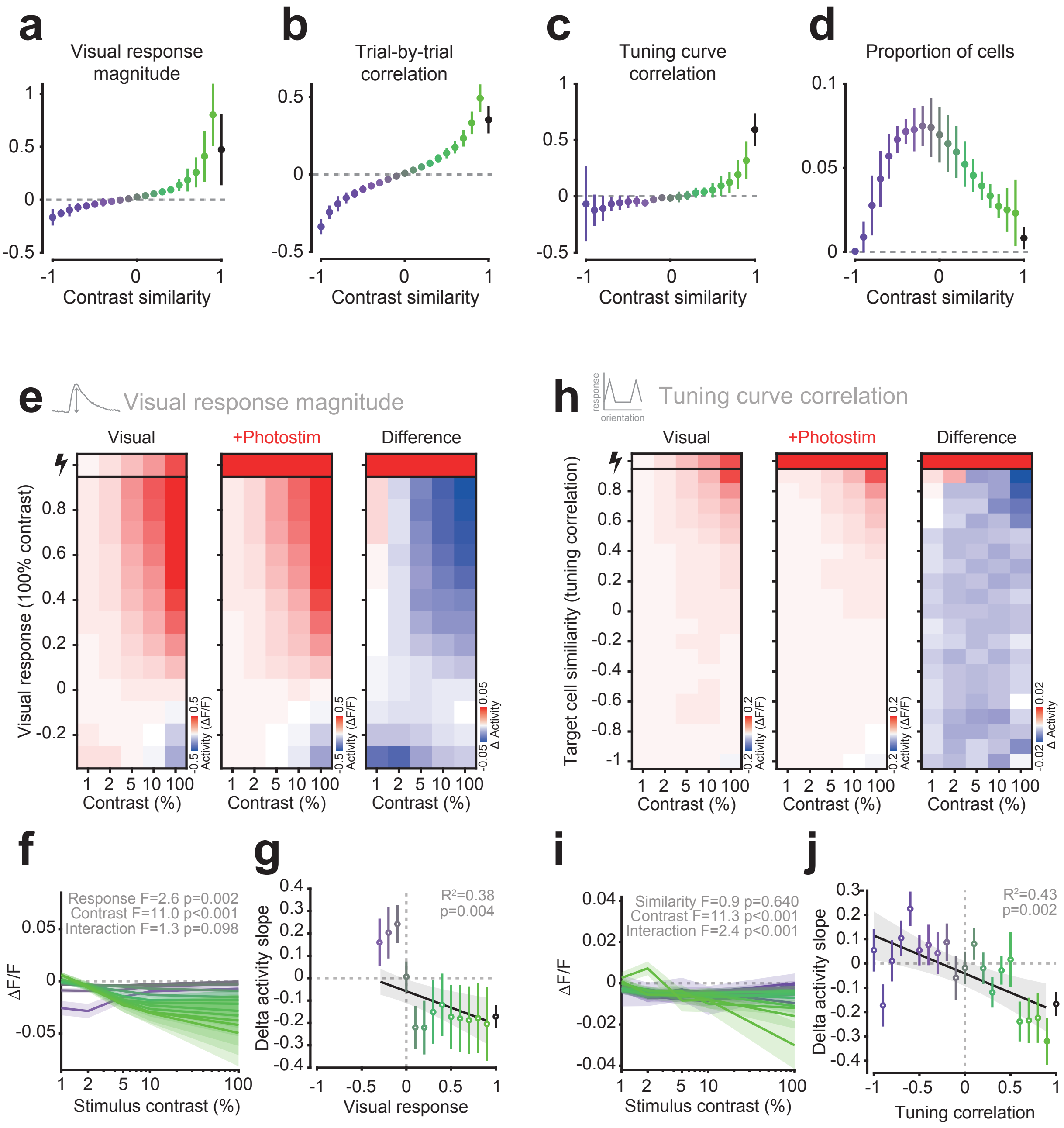

Supplementary Figure 7

Supplementary Figure 7. The mapping of contrast response similarity to other functional characterizations

a) Relating the contrast response similarity to visual response magnitudes (to 100% contrast). Neurons are binned according to their contrast curve similarity with the target group. Colors indicate contrast similarity (green = similar, purple = dissimilar) and the black datapoints at the far right indicate the directly stimulated targets. As neurons are more similar to the target neurons they respond more strongly to the visual stimulus. Error bars are SEM across session (n = 28).
b) Same as a) but relating contrast curve correlation to trial-by-trial correlation. We compute the Pearson correlation of all visual-only (0% to 100% contrasts) single trial responses across the session. As neurons are more similar to the target neurons in terms of average contrast-curves they are also more similar in the responses on single trials throughout the session.
c) Same as a) but relating contrast curve correlation to orientation tuning curves. As neurons are more similar to the target neurons in terms of contrast curves they are also more similar in terms of orientation tuning curves.
d) Showing the proportion of neurons per functional similarity bin.
e) Same as Fig. 3G but binning background neurons by visual response magnitude, measured on 100% visual stimuli trials (cross validated responses coming from half the trials not used for contrast similarity measurement). Not the dominant effect of suppression in the most strongly visually responsive neurons. Note the slight facilitation as low contrasts.
f) Same as Fig. 3H but binning background neurons by visual response magnitude. Lines represent the mean and shading represents SEM across session (n=28 sessions, 14 mice).
g) Same as Fig 3I but binning background neurons by visual response magnitude. Shading represent the CI of the fitted line.
h) Same as Fig. 3G but binning background neurons by the correlation of orientation tuning curves with the average target neurons tuning curve Orientation tuning curves are calculated from an initial mapping session where 8 orientations (all rewarded) were presented for 8 trials each in randomized order. Photostimulation responses are cross validated responses coming from half the trials not used for contrast similarity measurement. Note the non-specific suppression, but the strongest suppression is seen in the most similar neurons at high contrast. Note the slight facilitation at low contrasts.
i) Same as Fig. 3H but binning background neurons by the correlation of orientation tuning curves.
j) Same as Fig. 3I but binning background neurons by the correlation of orientation tuning curves.

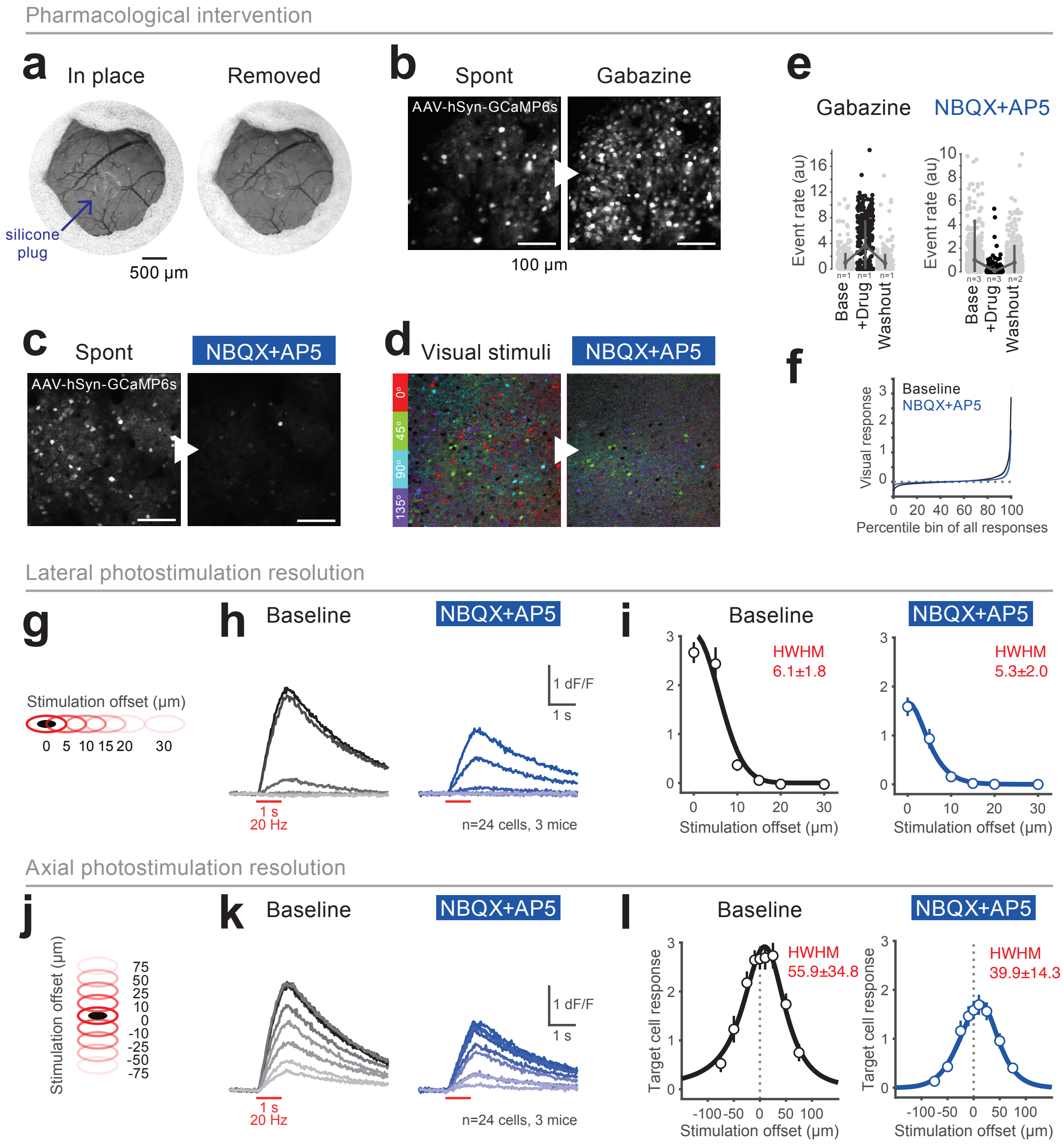

Supplementary Figure 8

Supplementary Figure 8. Physiological measurement of photostimulation resolution, with pharmacological intervention

a) We implanted chronic optical windows with small silicone-plugged holes in the center (Roome and Kuhn, 2014). These plugs could be removed at a later date providing pharmacological access to the imaged regions. We applied 1 mL droplet of drug-solution to the exposed brain surface for 15 minutes, before removing the droplet and re-immersing the objective with IVE solution and resume imaging.
b) Application of 1 mM Gabazine to block inhibitory currents results in an increase of spontaneous activity (summed deconvolved trace activity, a.u.).
c) Application of 1 mM NBQX and 2 mM AP5 to block excitatory currents (‘synaptic blockers’) results in a decrease of spontaneous activity (summed deconvolved trace activity, a.u.).
d) Co-application of synaptic blockers reduced the activity evoked by visual stimuli.
e) Quantification of the change in spontaneous event rate when Gabazine or NBQX/AP5 is applied.
f) Quantification of the reduction in visually-evoked responses with and without excitatory synaptic blockers applied.
g) Schematic showing the laterally-offset locations stimulated to quantify the photostimulation resolution curve of single cells (Same stimulation parameters as used in the rest of the study).
h) Average transients evoked by photostimulation of single cells. Pale colors indicate increasing lateral offsets. Blue traces are cells photostimulated after excitatory synaptic blockers were applied, these cells were in the same FOV but not explicitly matched to the cells stimulated before drug application.
i) Quantification of the photostimulation evoked responses as a function of lateral offset distance.
j) Same as g) but for axially offset stimulation locations.
k) Same as h) but for axially offset stimulation locations.
l) Same as i) but for axially offset stimulation locations.

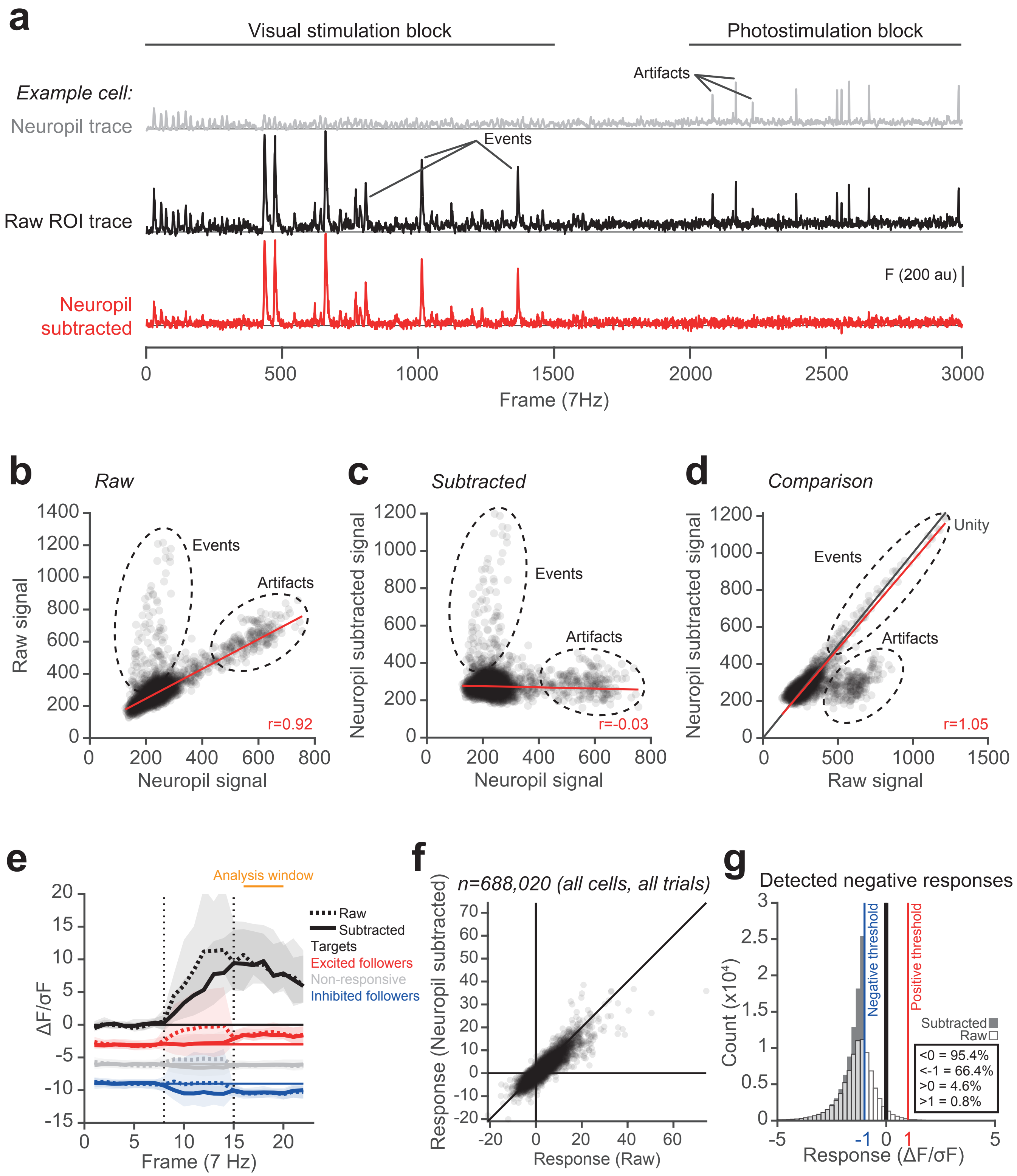
a
Visual stimulation block
Photostimulation block
Artifacts
Example cell:
Neuropil trace
Events
Raw ROI trace
F (200 au)
Neuropil
subtracted
Frame (7Hz)
b
Raw
Raw signal
Events
Artifacts
r=0.92
Neuropil signal
c
Subtracted
Neuropil subtracted signal
Events
Artifacts
r=-0.03
Neuropil signal
d
Comparison
Neuropil subtracted signal
Unity
Events
Artifacts
r=1.05
Raw signal
e
Analysis window
ΔF/σF
Raw
Subtracted
Targets
Excited followers
Non-responsive
Inhibited followers
Frame (7 Hz)
f
n=688,020 (all cells, all trials)
Response (Neuropil subtracted)
Response (Raw)
g
Detected negative responses
Count (x10⁴)
Negative threshold
Positive threshold
Subtracted
Raw
<0 = 95.4%
<-1 = 66.4%
>0 = 4.6%
>1 = 0.8%
Response (ΔF/σF)

Supplementary Figure 9. Minimal effects of neuropil subtraction on the measured photostimulation response magnitude and detection of negative responses

a) Example neuropil, ROI and neuropil-subtracted ROI traces from one cell. The segment of activity includes part of the orientation preference mapping block and the beginning of the photostimulation during behavior block. Example photostimulation artifacts are indicated.

b) The correlation between the neuropil and the raw ROI signal (each point represents the average of 7 frames). The slope of the robust-regression fit line is used to scale the neuropil signal before subtracting it from the ROI signal. Dashed circles indicate both the set of points roughly corresponding to photostimulation artifacts (shared between the ROI and the neuropil) and ROI events (unique to the ROI, not seen in the neuropil).

c) The correlation between the neuropil and the neuropil-subtracted ROI signal.

d) The correlation between the raw ROI and the neuropil-subtracted ROI signal.

e) Extracted photostimulus-triggered traces for all cells in one example experiment on one example trial. Solid line indicates the median across the cells in that response category (targeted, excited, non-responsive, inhibited). Shaded region indicates interquartile range. Dotted vertical lines indicate the photostimulation period, note this is the period affect by the photostimulation artifact and is the region with obvious difference between raw and neuropil subtracted traces. Responses are extracted in a window after photostimulation offset to avoid the artefact contamination.

f) Pooling all responses from all cells on all trials (to CT stimulation alone) in all sessions, comparing with and without neuropil subtraction.

g) Assessing the detected negative responses in the final, neuropil subtracted traces. All have a response of less than 1 $\Delta F/\sigma F$, as per criteria (black histogram). 95.4% of these negative responses are associated with a negative response even when not performing neuropil subtraction (blue histogram).

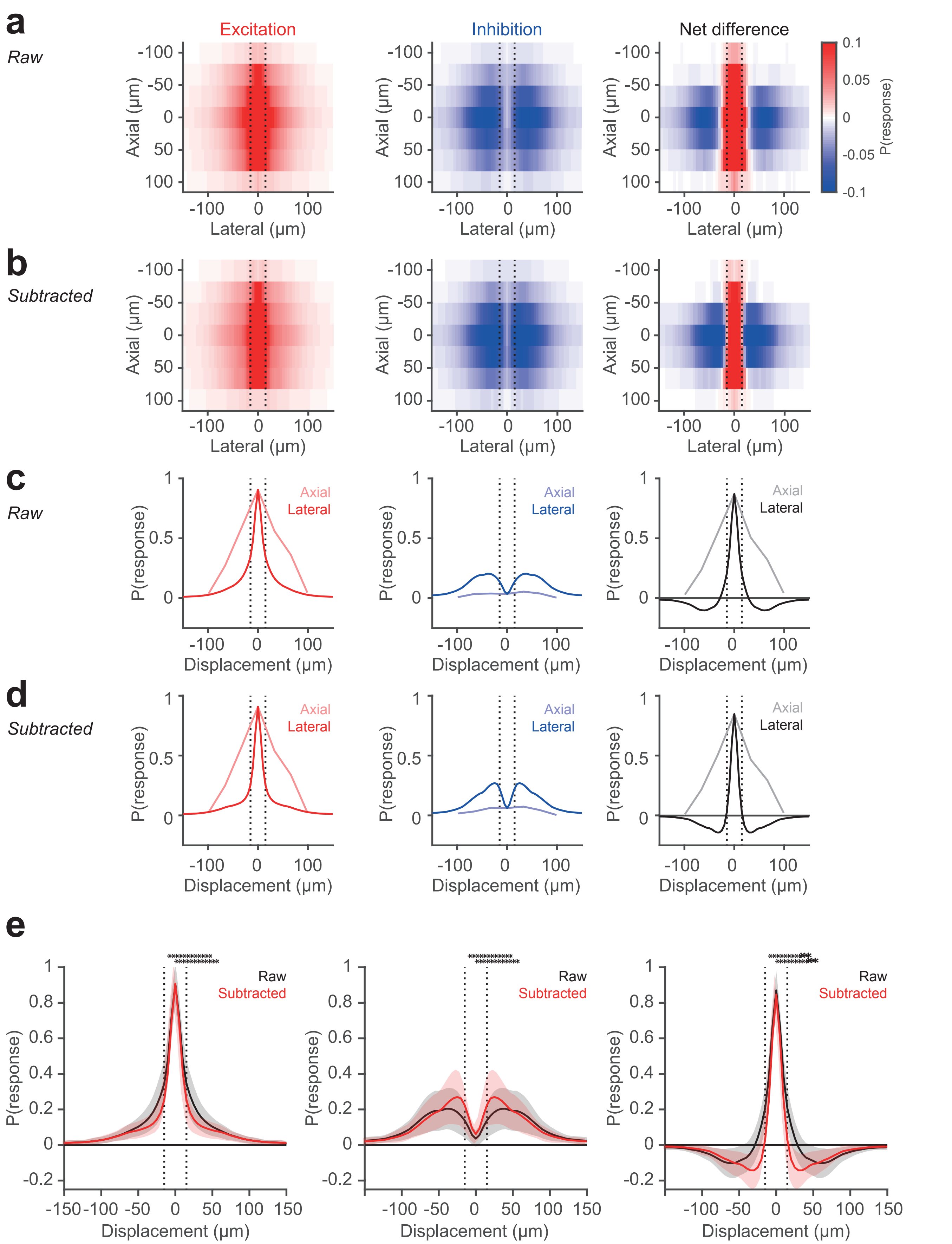

Supplementary Figure 10

Supplementary Figure 10. Minimal effects of neuropil subtraction on the spatial profile of the network response to photostimulation

a) The trial-averaged P(response) for excitation (left) inhibition (middle) and the difference between the two (right) for all cells, including directly targeted cells, binned spatially and aligned to the nearest target cell (0 μm lateral, 0 μm axial). The dashed vertical lines indicate the volume (30 μm diameter cylinder extending through all axial planes) excluded for 'follower' analysis in the manuscript. P(response) metrics calculated on raw, non-subtracted, ROI traces. Data at negative lateral displacements are the same as for positive displacements, for display purposes only.
b) Same as a) but for the final neuropil subtracted traces.
c) The average curves of P(response) against distance from nearest target for the lateral dimension at the 0 μm axial plane, and for the axial dimension at the 0 μm lateral position. Left: excitatory response, middle: inhibitory responses, right: the difference between the two. P(response) is calculated on the raw, non-subtracted, ROI traces.
d) Same as for c) but for the final neuropil subtracted traces.
e) Comparison of the lateral P(response) curves seen in c) and d) with and without neuropil subtraction. Shaded region indicates the standard deviation across all stimulation sessions. Left: excitatory response, middle: inhibitory responses, right: the difference between the two. (N = 63 sessions, 14 mice. ** denotes $P < 0.01$, Wilcoxon signed rank test with Bonferroni correction).
