# Supplementary material for: The influence of cortical activity on perception depends on behavioral state and sensory context
Source: Nat Commun. 2024 Mar 19;15:2456. doi: 10.1038/s41467-024-46484-5 (PMC10951313; doi:10.1038/s41467-024-46484-5)
Supplement: Supplementary file 3 — Reporting Summary [file 41467_2024_46484_MOESM3_ESM.pdf]

nature portfolio

Corresponding author(s): Michael Hausser

Last updated by author(s): Dec 18, 2023

Reporting Summary

Nature Portfolio wishes to improve the reproducibility of the work that we publish. This form provides structure for consistency and transparency in reporting. For further information on Nature Portfolio policies, see our Editorial Policies and the Editorial Policy Checklist.

Statistics

For all statistical analyses, confirm that the following items are present in the figure legend, table legend, main text, or Methods section.

n/a	Confirmed	
☐	☒	The exact sample size (*n*) for each experimental group/condition, given as a discrete number and unit of measurement
☐	☒	A statement on whether measurements were taken from distinct samples or whether the same sample was measured repeatedly
☐	☒	The statistical test(s) used AND whether they are one- or two-sided<br>*Only common tests should be described solely by name; describe more complex techniques in the Methods section.*
☐	☒	A description of all covariates tested
☐	☒	A description of any assumptions or corrections, such as tests of normality and adjustment for multiple comparisons
☐	☒	A full description of the statistical parameters including central tendency (e.g. means) or other basic estimates (e.g. regression coefficient) AND variation (e.g. standard deviation) or associated estimates of uncertainty (e.g. confidence intervals)
☐	☒	For null hypothesis testing, the test statistic (e.g. *F*, *t*, *r*) with confidence intervals, effect sizes, degrees of freedom and *P* value noted<br>*Give P values as exact values whenever suitable.*
☒	☐	For Bayesian analysis, information on the choice of priors and Markov chain Monte Carlo settings
☒	☐	For hierarchical and complex designs, identification of the appropriate level for tests and full reporting of outcomes
☐	☒	Estimates of effect sizes (e.g. Cohen's *d*, Pearson's *r*), indicating how they were calculated

Our web collection on statistics for biologists contains articles on many of the points above.

Software and code

Policy information about availability of computer code

Data collection	Simultaneous two-photon optogenetics and calcium imaging data were collected using PrairieView (Bruker), Blink (v1.1.3.528, Meadowlark) and custom-written MATLAB (2015a-2019b, Mathworks) code for SLM calibration and photostimulation pattern production. PsychoPy was used for visual stimulation and LabVIEW (2017, National Instruments) or MATLAB were used for post-hoc synchronization. Custom code used for data acquisition, photostimulation control, behavioral training and analysis have been deposited online<br>Naparm https://github.com/llerussell/Naparm DOI: 10.5281/zenodo.10449686<br>PyBehaviour https://github.com/llerussell/PyBehaviour DOI: 10.5281/zenodo.10449684<br>3D SLM calibration https://github.com/llerussell/SLMTransformMaker3D DOI: 10.5281/zenodo.10449682<br>STAMovieMaker https://github.com/llerussell/STAMovieMaker DOI: 10.5281/zenodo.10449680<br>RawDataStream https://github.com/llerussell/Bruker_PrairieLink DOI: 10.5281/zenodo.10449690<br>Objective rotation https://github.com/llerussell/MONPangle DOI: 10.5281/zenodo.10449688
Data analysis	Calcium imaging registration and segmentation was performed using Suite2P (Pachitariu et al 2017). Analysis was performed using custom written MATLAB (2015a-2019b, Mathworks) code. Statistical comparisons were performed using built-in Matlab functions

For manuscripts utilizing custom algorithms or software that are central to the research but not yet described in published literature, software must be made available to editors and reviewers. We strongly encourage code deposition in a community repository (e.g. GitHub). See the Nature Portfolio guidelines for submitting code & software for further information.

Data

Policy information about availability of data

All manuscripts must include a data availability statement. This statement should provide the following information, where applicable:

- Accession codes, unique identifiers, or web links for publicly available datasets
- A description of any restrictions on data availability
- For clinical datasets or third party data, please ensure that the statement adheres to our policy

Datasets supporting the findings of the study are available from the corresponding author on request.

Research involving human participants, their data, or biological material

Policy information about studies with human participants or human data. See also policy information about sex, gender (identity/presentation), and sexual orientation and race, ethnicity and racism.

Reporting on sex and gender	NA
Reporting on race, ethnicity, or other socially relevant groupings	NA
Population characteristics	NA
Recruitment	NA
Ethics oversight	NA

Note that full information on the approval of the study protocol must also be provided in the manuscript.

Field-specific reporting

Please select the one below that is the best fit for your research. If you are not sure, read the appropriate sections before making your selection.

☒ Life sciences ☐ Behavioural & social sciences ☐ Ecological, evolutionary & environmental sciences

For a reference copy of the document with all sections, see nature.com/documents/nr-reporting-summary-flat.pdf

Life sciences study design

All studies must disclose on these points even when the disclosure is negative.

Sample size	As part of the review process we collected an entire new dataset that supplants the original dataset. The new, second dataset is designed to more directly target the findings of the first with more clarity by disambiguating a couple of factors. The second dataset therefore replicates and validates the first. Results from only the first dataset are not available in the manuscript but remain available as a preprint on biorXiv (https://doi.org/10.1101/706010). No power analysis or other statistical methods were used to pre-determine sample sizes but our sample sizes were similar to those in previous publications (Carrilo-Reid et al 2019, Marshel et al 2019, Chettih and Harvey 2019, Dalgleish et al 2020). No additional data was collected after the reported statistical result was obtained. All statistical tests were two sided unless indicated in the Methods.
Data exclusions	We excluded experiment trials if >50% of photostimulation targets failed to respond on that trial. We also excluded trials if the mice licked early (within the first 150 ms of the presentation of the visual stimulus). Whole sessions were then excluded if fewer than 10 trials in any trial type remained (the median minimum number of trials per trial type (note each session has 12 trial types) in included sessions = 31 trials (range 10–56)).Out of 32 completed sessions, 3 were excluded because of poor photostimulation efficiency
Replication	All figures involved experimental series using multiple mice and reported results held across mice. No attempt at replication was made outside of the reported results.
Randomization	Randomization of animals to different groups is not relevant to our study as all mice used in individual experimental series had the same genotype and rearing conditions. Behavioural stimulus (visual and photostimulation) delivery was pseudorandomized in time and order.
Blinding	Investigators were not blinded to group allocation. No group division of animal is relevant to this study.

Reporting for specific materials, systems and methods

We require information from authors about some types of materials, experimental systems and methods used in many studies. Here, indicate whether each material, system or method listed is relevant to your study. If you are not sure if a list item applies to your research, read the appropriate section before selecting a response.

Materials & experimental systems

n/a	Involved in the study
☒	☐ Antibodies
☒	☐ Eukaryotic cell lines
☒	☐ Palaeontology and archaeology
☐	☒ Animals and other organisms
☒	☐ Clinical data
☒	☐ Dual use research of concern
☒	☐ Plants

Methods

n/a	Involved in the study
☒	☐ ChIP-seq
☒	☐ Flow cytometry
☒	☐ MRI-based neuroimaging

Animals and other research organisms

Policy information about studies involving animals; ARRIVE guidelines recommended for reporting animal research, and Sex and Gender in Research

Laboratory animals	Male and female adult mice (P49-P67 for surgeries, P77-P171 for experiments) were used. The following genotypes were used: TITL-GCaMP6s (Jax #024104). Animals were kept at a normal 12hr light/dark cycle at a temperature of 22oC and 62% humidity.
Wild animals	No wild animals were used in this study.
Reporting on sex	Male and female mice were used in equal proportion dependent on availability through in house bred litters. Sex was not a factor in experimental design.
Field-collected samples	No samples were collected in the field
Ethics oversight	All experimental procedures were carried out under license from the UK Home Office in accordance with the UK Animals (Scientific Procedures) Act (1986).

Note that full information on the approval of the study protocol must also be provided in the manuscript.

Plants

Seed stocks	NA
Novel plant genotypes	NA
Authentication	NA
